# Clinical, neurophysiological evaluation and genetic features of axonal Charcot–Marie–Tooth disease in a Chinese family

**DOI:** 10.3389/fneur.2023.1337065

**Published:** 2024-02-02

**Authors:** Li Cao, Jie Yang, Xiaohuan Zhang, Xu Wang, Zhangyuwei Chen, Song Tan, Jiyun Yang

**Affiliations:** ^1^Sichuan Provincial Key Laboratory for Human Disease Gene Study, Center of Medical Genetics, Sichuan Provincial People’s Hospital, School of Medicine, University of Electronic Science and Technology of China, Chengdu, China; ^2^Research Unit for Blindness Prevention of Chinese Academy of Medical Sciences (2019RU026), Sichuan Academy of Medical Sciences, Chengdu, China; ^3^Department of Neurology, Sichuan Provincial People’s Hospital, School of Medicine, University of Electronic Science and Technology of China, Chengdu, China

**Keywords:** Charcot–Marie–Tooth disease (CMT), the mitochondrial transfer RNA (*mt-tRNA*^*val*^) gene, the m.1661A>G variant, neuroelectrodiagnostic testing, peripheral neuropathies

## Abstract

Charcot–Marie–Tooth disease (CMT) is a group of inherited peripheral neuropathies related to variants in the mitochondrial transfer RNA (*mt-tRNA*^*val*^) gene. Here, we report a Chinese family harboring the m.1661A>G variant in the *mt-tRNA*^*val*^ gene. Clinical evaluation, neuroelectrodiagnostic testing, and nerve biopsy were performed on four affected family members. Weakness, spasms, and pain in the limbs (especially in the lower limbs) were the main complaints of the proband. Physical examination revealed atrophy and weakness in the distal limbs, increased muscle tone, and hyperreflexia in four limbs. Neuroelectrodiagnostic tests and nerve biopsy supported an axonal polyneuropathy. This study furthers the understanding of phenotype diversity caused by variants in the *mt-tRNA*^*val*^ gene in CMT.

## Introduction

Charcot–Marie–Tooth disease (CMT) is a group of inherited peripheral neuropathies characterized by motor and sensory symptoms (1, 2). The onset is usually in childhood or early adulthood, the typical clinical features are distal muscle weakness and atrophy (3). Based on neurophysiological examination, CMT can be divided into three forms: (1) demyelinating form caused by Schwann cell dysfunction, (2) axonal form caused by degeneration of peripheral nerve axons, and (3) intermediate form (4–6).

Most CMT are found to be monogenic disease, and genetic patterns include autosomal dominant, autosomal recessive, X-linked and maternal (mitochondrial) inheritance (7). In recent years, more than 100 genes have been identified as genetic causes of CMT due to the rapid development of next generation sequencing (NGS) technologies (1, 8, 9). However, there are few reports on mitochondrial mutations related to CMT.

Mitochondria are a crucial organelle in nerve cells, whose primary function is to produce ATP and provide energy for various cellular activities. Due to the long axons, peripheral nerves have peculiar energetic requirements to maintain complex metabolic mechanisms. Therefore, correct mitochondrial function is crucial for maintaining energy metabolism. Many subtypes of CMT are associated with abnormality in mitochondrial function. Pathogenic mutations in the *mt-tRNA*^*val*^ gene may impair mitochondrial translation, leading to CMT (10, 11).

Here, we report the variant of m.1661A>G in a Chinese family with CMT with a mitochondrial inheritance pattern. Furthermore, we describe in detail the clinical and neurophysiological studies data and nerve biopsy findings of the recruited family members. This study furthers the understanding of phenotype diversity caused by variants in the *mt-tRNA*^*val*^ gene in CMT.

## Materials and methods

### Participants and neurophysiological examination

The research was approved by the Ethics Committee of Sichuan Provincial People’s Hospital. All participants signed the informed consent and agreed to undergo a detailed clinical examination, including assessment of medical history and physical examination performed by neurologists.

All participants underwent standard neurophysiological examination via Sierra Summit (Cadwell industries, Washington, America). Sensory conduction velocity, sensory nerve action potential (SNAP), motor conduction velocity (MCV), compound muscle action potential (CMAP), distal motor latency (DML), and F-wave were recorded from median, ulnar, peroneal, and tibial nerves. Electromyography (EMG) was performed on the distal and proximal muscles of the limbs.

### Sural nerve biopsy analysis

Hematoxylin–eosin staining and pathological microscopes were used to examine the proband’s sural nerves. Immunohistochemistry staining was performed to examine the expression of human leukocyte antigen DR (HLA-DR), myelin basic protein (MBP), leukocyte common antigen (LCA), and KP1. Images were captured at a magnification of 40×.

### Genetic analyses

Whole genome sequencing (WGS) was performed on two affected members (IV:2 and III:1) and one unaffected member (IV:1) via the Illumina platform (CIPHER, Beijing, China). Then, Sanger sequencing was used to validate the candidate variants and co-segregation between the genotype and the CMT phenotype in the family.

## Results

### Pedigree

**Figure 1:**
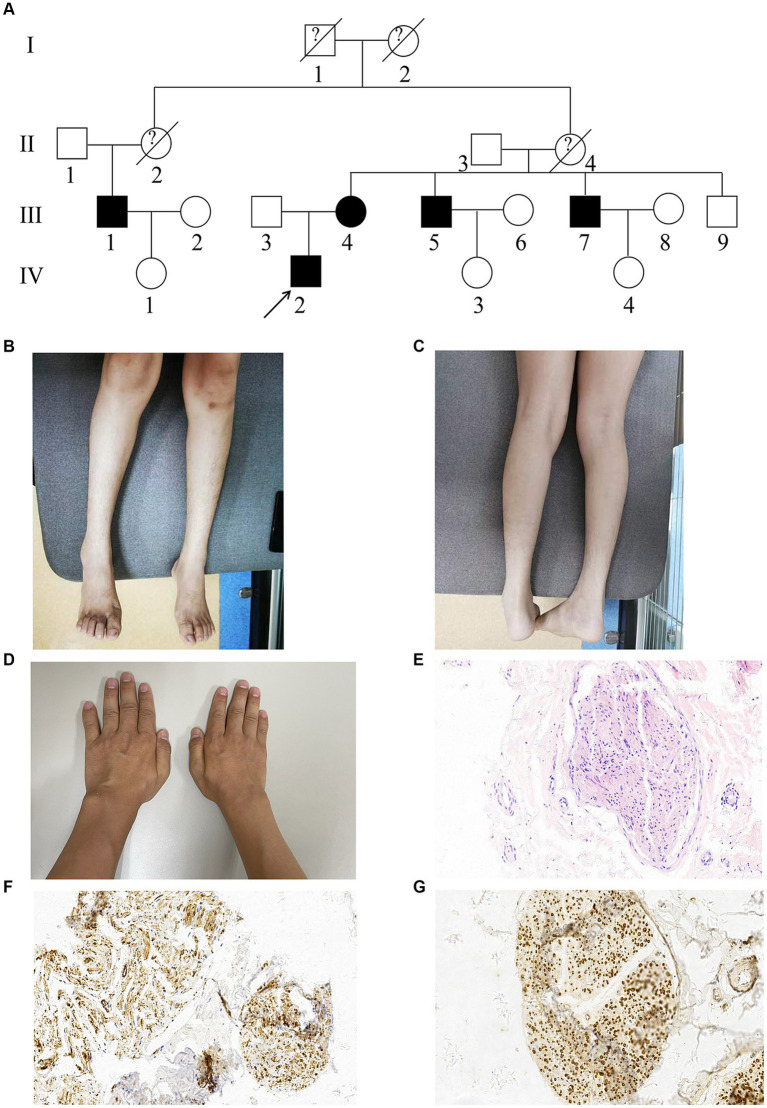
Pedigree, clinical and nerve biopsy findings of the proband (IV:2). **(A)** Pedigrees of the CMT family. Affected individuals are denoted in Black. The arrow indicates the proband. **(B,C)** The proband exhibited obvious atrophy in tibialis anterior and gastrocnemius of lower limbs. **(D)** The proband exhibited moderate atrophy in dorsal interosseous muscles of upper limbs. **(E)** Hematoxylin–eosin staining (HE staining) of the proband’s sural nerves. **(F,G)** Immunohistochemistry showed HLA-DR (positive) and MBP (positive).

A Chinese family with CMT was recruited (Figure 1A). Participants included five affected members (III:1, III:4, III:5, III:7, and IV:2) and four unaffected members (III:3, IV:1, IV:3, and IV:4). All descendants of male members with the CMT phenotype were unaffected.

### Clinical investigations

#### Patient 1 (proband, IV:2)

Patient 1 was a 36 years-old man. At 20 years of age, he complained of weakness, pain, and spasms in his lower limbs after walking or sitting for a long time. Later, his sleep quality was affected by continuous spasms and pain, and he woke more than a dozen times every night. He was also diagnosed with hereditary spastic paraplegia and was treated with baclofen. Since the age of 33, the pain in the outer and posterior sides of the right leg, buttock, and coccyx has progressively worsened and caused a severe sleep disorder. Finally, after a nerve conduction study (NCS), EMG and sural nerve biopsy, he was diagnosed with hereditary motor and sensory neuropathy type 2.

Neurological examination revealed obvious atrophy in the tibialis anterior and gastrocnemius of the lower limbs (Figures 1B,C), moderate atrophy in the dorsal interosseous muscles of the upper limbs (Figure 1D), increased muscle tone, weakness in the four distal limbs through manual muscle tests (MMT 3/5 in the lower limbs and 4/5 in the upper limbs), and hyperreflexia in the four limbs. Pinprick sensations were symmetrically decreased in the distal lower limbs.

NCS (Table 1) showed reduced amplitude of CMAPs in the bilateral tibial, peroneal, and right ulnar nerves. There was a slight slowing of MCVs in the left peroneal nerve and some prolonging of DMLs in bilateral tibial nerves and the left peroneal nerve. SNAPs could not be evoked in any sampled nerve. F-wave could not be evoked in the right tibial nerve, and F-wave latency was extended in the left tibial nerve.

**Table 1:** Electrophysiological data.

Nerve patient	Patient 1 (IV:2)		Patient 2 (III:4)		Patient 3 (III:5)		Patient 4 (III:7)	
	Left	Right	Left	Right	Left	Right	Left	Right
**Median nerve**								
MCV (m/s)	55	59	45	50	56	54	55.1	59.4
Distal latency (ms)	3.33	3.44	4.58	4.06	3.70	3.49	3.1	3.5
CMAP amplitude (mV)	11.8	7.9	10.9	10.9	13.2	16.9	10.5	10.7
SCV (m/s)	NR	NR	NR	NR	NR	NR	52.2	55
SNAP amplitude	NR	NR	NR	NR	NR	NR	20.6	14.9
F mean latency (ms)	28.1	29.5	31.2	NR	25.9	25.8	24.9	/
F%	100	90	30	0	100	100	100	/
**Ulnar nerve**								
MCV (m/s)	59	55	35	46	65	52	60.9	60.5
Distal latency (ms)	3.23	3.02	2.81	3.54	3.54	3.23	2.7	2.8
CMAP amplitude (mV)	7.8	6.4	3.5	5.2	8.6	4.4	10.8	13.4
SCV (m/s)	NR	NR	NR	NR	NR	NR	54.8	56.2
SNAP amplitude	NR	NR	NR	NR	NR	NR	12.9	13.8
F mean latency (ms)	30.7	30.0	30.1	30	27.7	27.8	49.3	49.7
F%	100	80	60	90	100	80	100	100
**Tibial nerve**								
MCV (m/s)	43	46	NR	NR	35	NR	48.9	48.3
Distal latency (ms)	6.25	4.58	NR	NR	7.60	NR	4.0	4.0
CMAP amplitude (mV)	4.6	2.0	NR	NR	0.6	NR	12.7	16.0
**Sural nerve**								
SCV (m/s)	NR	NR	NR	NR	NR	NR	56.3	51.2
SNAP amplitude	NR	NR	NR	NR	NR	NR	5.6	2.1
**Superficial peroneal nerve**								
SCV (m/s)	NR	NR	NR	NR	NR	NR	42.9	44.5
SNAP amplitude	NR	NR	NR	NR	NR	NR	4.9	2.5

MCV, motor conduction velocity; CMAP, compound motor action potential; SCV, sensory conduction velocity; SNAP, sensory nerve action potential; NR, not recordable.

EMG revealed chronic neurogenic lesions with reinnervated MUAPs and decreased recruitment in the right tibialis anterior, left quadriceps medialis, first interosseous dorsal muscle, and right biceps.

### Sural nerve biopsy

The biopsy of the six bundles of sural nerves from the proband showed a medium depletion of myelinated nerve fibers, especially of the large-diameter fibers. Schwann cells exhibited mild proliferation with degeneration. Some fiber axonal microfilaments increased and aggregated, while a few unmyelinated fiber axonal microfilaments degenerated. Immunohistochemistry (Figures 1E–G) showed that the samples were positive for HLA-DR and MBP and negative for LCA and KP1. These results suggested that the histopathological profile of the patient was mainly axonal degeneration with nerve damage.

#### Patient 2 (III:4)

Patient 2 was a 59 years-old woman who was the mother of the proband. At an age of around 18 years, she began to feel slight pain in her knee, which she initially thought was due to physical labor; however, the pain gradually worsened over time. She also complained of weakness in her lower limbs.

NCS showed decreased CMAPs in bilateral ulnar nerves. CMAPs could not be evoked in any nerve of the lower limbs, and SNAPs could not be evoked in any sampled nerve. F-wave could not be evoked in the right median nerve, and F-wave latencies extended in the nerves of the upper limbs.

#### Patient 3 (III:5)

Patient 3 was a 58 years-old man who was the uncle of the proband. At the age of around 20 years, he began to feel weakness after long-distance walking and climbing hills. The symptoms gradually and slowly worsened. Until the age of 40, he felt very weak when walking, but did not have any pain.

Similar to his nephew and elder sister, SNAPs could not be evoked in any sampled nerve, and CMAPs could not be evoked in the nerves of the lower limbs. CMAPs were decreased in the left tibial and bilateral ulnar nerves. F-waves could not be evoked in bilateral tibial nerves.

The EMGs of two patients (III:4 and III:5) showed chronic neurogenic damage to the muscles of the upper and lower limbs. The results of three patients (III:4, III:5, and IV:2) indicated a length-dependent motor and sensory axonal polyneuropathy.

#### Patient 4 (III:7)

Patient 4 (proband’s uncle) was a 56 years-old man who did not complain of any weakness or pain. However, Sanger sequencing revealed that he carried the variant m.1661A>G. It was unclear why the patient did not show any symptoms of the disease. Therefore, NCS was performed on him, which revealed a slight impairment of the sensory fibers of the peripheral nerves in his four limbs, particularly in the lower limbs.

### Genetic findings

WGS sequencing of mtDNA was performed on three family members (IV:2, III:1 and IV:1). Patients IV:2 and III:1, but not patient IV:1, carried the genes, which were filtered to identify a potential causal mutation. Considering a mitochondrial inheritance pattern, genes located in mitochondria were focused on. Finally, a homoplasmic variant (m.1661A>G) in the *mt-tRNA*^*val*^ gene was identified in the proband (IV:2) and proband’s uncle (III:1) using WGS.

**Figure 2:**
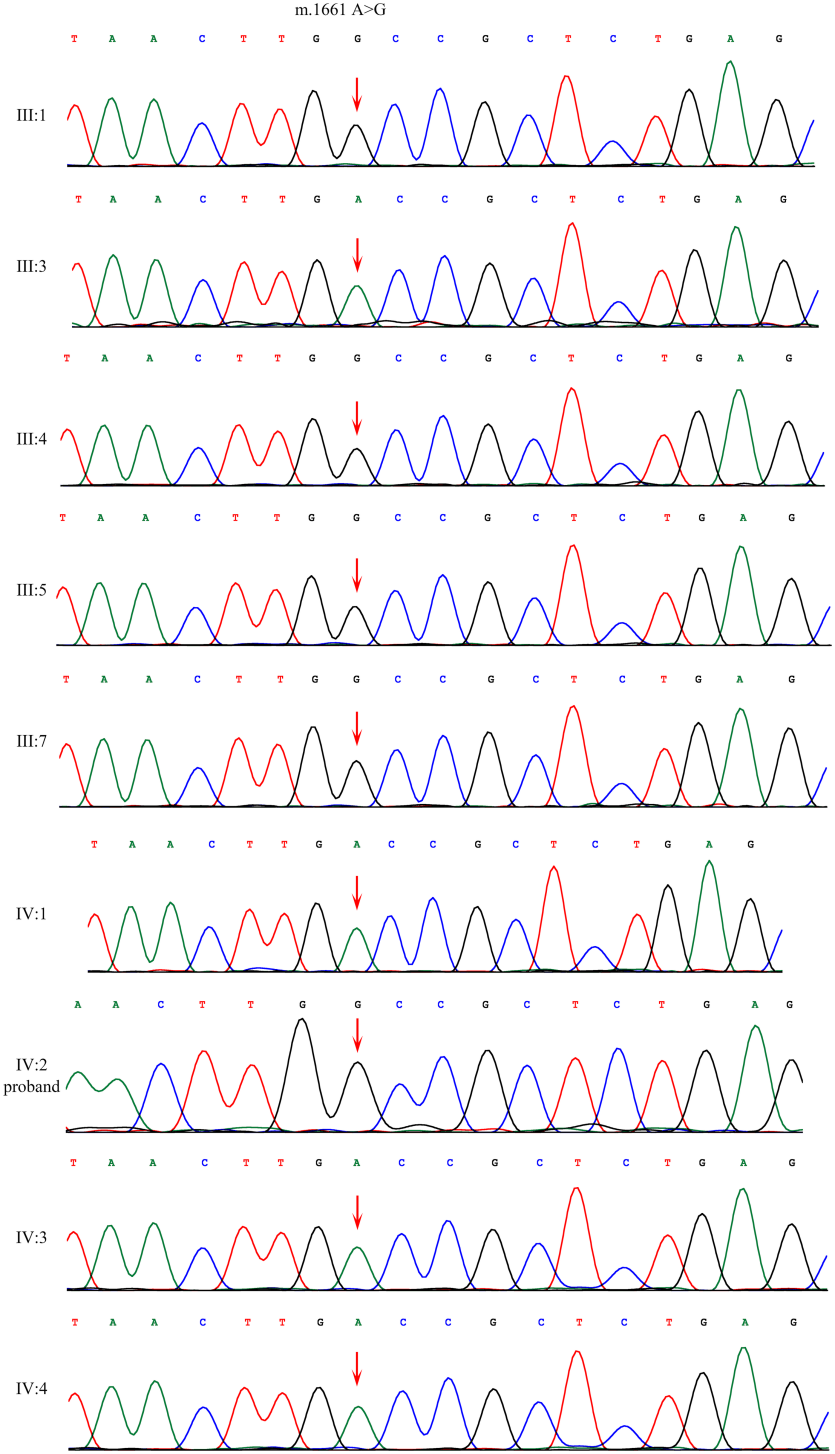
Sanger sequencing of the m.1661A>G variant in *mt-tRNA*^*val*^ gene showed that the variant were present among patients and absent among normal individuals.

Sanger sequencing showed that the homoplasmic variant: m.1661 A>G was identified among four affected family members (III:1, III:4, III:5, IV:2) and one family member that denied any symptoms (III:7). This variant is absent in four unaffected individuals (III:3, IV:1, IV:3, IV:4) (Figure 2).

## Discussion

Here, we reported a Chinese family with a length-dependent motor and sensory neuropathy based on axonal lesions and shown that the family members harbored the m.1661A>G variant in the *mt-tRNA*^*val*^ gene. Weakness of the limbs, especially the lower limbs, was the main symptom of most of the patients. In addition, continuous severe spasms and pain in the lower limbs were the other symptoms in this family, especially in the proband, which was different from the previous reports.

Although there were indeed some cases who presented Spastic paresis and Babinski reflex in the previously reported family from Venezuela (12), the damage of pyramidal tracts seems to be more prominent (severe spasmodic paresis and pain, which requires long-term use of a baclofen pump to relieve) in our study especially in the proband, and leading to clinical diagnosis of HSP in different hospitals. However, genetic tests either for HSP or for CMT2 did not show any positive results. Then, pedigree analysis of this family showed a mitochondrial inheritance pattern, and m. 1661A>G variant in the *mt-RNA*^*val*^ gene via WGS sequencing was identified among affected family members, which can also explain the phenotypic heterogeneity in this family. Given the severity of peripheral nerve and vertebral tract damage in this family, it may be more appropriate to refer to it as a complex neurological syndrome with overlap between spinal paresis and axial sensorimotor polyneuropathy. Electrodiagnostic results indicated significant axonal lesions in four limbs, especially in the lower limbs, which were confirmed by decreased or absent CMAPs and SNAPs, while conduction velocities and distal latencies were normal or just slightly slowing. Sensory fibers were more severely damaged than motor fibers. EMG showed reduced recruitment and reinnervated MUAPs without any abnormal spontaneous potential, indicating a chronic stage of axonal loss lesions. The sural nerve biopsy of the proband showed axonal degeneration with nerve damage, which was consistent with electrodiagnostic results.

Notably, patient 4 (III:7) denied any symptoms of sensation and movement in the limbs. However, his genetic and neurophysiological examination showed abnormalities. He carried the variant m.1661A>G and showed a slight impairment of the sensory fibers of the peripheral nerves in his four limbs, particularly in the lower limbs. The subtle nature of the changes, according to the EMG, may explain why the patient denied having any symptoms.

To identify the gene related to phenotype, we considered modes of inheritance other than mitochondrial transmission, but no candidate mutations that that segregate with the disease phenotype have been identified, through WGS of two affected members (IV:2 and III:1) and one unaffected member (IV:1). All descendants of male members with the CMT phenotype were unaffected, which led us to focus on mitochondrial genetics. Finally, the m.1661A>G variant in the *mt-tRNA*^*val*^ gene was identified, which is homoplasmic in blood. The main function of *mt-tRNA*^*val*^ is to bring charged valine into the ribosome, in order to translate the valine codon during mitochondrial protein synthesis. In addition, it is also a structural component of the human mitochondrial ribosome (13). The m.1661A>G mutation is in the stem region of the tRNA T-loop, which may interfere with mitochondrial function in any cell with high energy metabolism. This may explain the reason why *mt-tRNA*^*val*^ mutations lead to CMT.

Our study indicated that the phenotypes of *tRNA*^*val*^-related CMT are complicated and diverse. The variable and overlapping clinical phenotypes between syndromes may explain the proband’s past clinical diagnosis. These Chinese family members were unable to receive proper medical care for many years due to the complexity of the diagnosis, and they underwent many genetic tests. Finally, they were diagnosed with CMT caused by the homoplasmic m.1661A>G variant in the *mt-tRNA*^*val*^ gene via WGS sequencing. This variant was previously reported in a Venezuelan family (12). To the best of our knowledge, this study is the first to report this variant in a Chinese family. The study expands the phenotype diversity and furthers the understanding of CMT caused by mitochondrial gene mutations in different ethnic groups.

## Data availability statement

The original contributions presented in the study are included in the article/Supplementary material, further inquiries can be directed to the corresponding author.

## Ethics statement

The studies involving humans were approved by the Ethics Committee of Sichuan Provincial People’s Hospital. The studies were conducted in accordance with the local legislation and institutional requirements. The participants provided their written informed consent to participate in this study. Written informed consent was obtained from the individual(s) for the publication of any potentially identifiable images or data included in this article.

## Author contributions

LC: Investigation, Methodology, Validation, Writing – original draft. JeY: Investigation, Methodology, Validation, Writing – original draft. XZ: Data curation, Writing – original draft. XW: Data curation, Writing – original draft. ZC: Data curation, Writing – original draft. ST: Project administration, Resources, Supervision, Writing – review & editing. JnY: Project administration, Resources, Supervision, Writing – review & editing.
