# Supplementary material for: Clinical, neurophysiological evaluation and genetic features of axonal Charcot–Marie–Tooth disease in a Chinese family
Source: Front Neurol. 2024 Feb 2;14:1337065. doi: 10.3389/fneur.2023.1337065 (PMC10870769; doi:10.3389/fneur.2023.1337065)
Supplement: Supplementary file 1 [file Table_1.doc]

**Supplementary table**

| Patient | Gender | Age  (ys) | Age of onset(ys) | Phenotype | mt-tRNA^val^  mutation | CMT classification according to MCV of the ulnar nerve |
| --- | --- | --- | --- | --- | --- | --- |
| IV:2  (proband) | male | 36 | 20 | Weakness of lower limbs，  Spastic paresis with severe pain | m.1661A>G | axonal CMT |
| III:1 | male | 60 | 40 | CMT | m.1661A>G | - |
| III:3 | male | 67 | - | normal | - | - |
| III:4 | female | 59 | 18 | weakness of lower limbs，  Spastic paresis with pain | m.1661A>G | axonal CMT |
| III:5 | male | 58 | 20 | weakness of lower limbs | m.1661A>G | axonal CMT |
| III:7 | male | 56 | - | no complaint | m.1661A>G | sightly subclinical sensory neuropathy |
| IV:1 | female | 35 | - | normal | - | - |
| IV:3 | female | 35 | - | normal | - | - |
| IV:4 | female | 30 | - | normal | - | - |

CMT: Charcot-Marie-Tooth
